# Cloroform in Surgical Operations

**Published:** 1856-07

**Authors:** 


*Chloroform in Surgical Operations—the cases in which it should not be applied.* — Mr. Coulson, in a discussion at the Medical Society of London, October 13th last, said, that all surgeons in large practice must have employed this agent a great number of times, and experience has enabled them to determine several points about which there can be, he thought, no question any longer. In the first place, it may be considered settled that chloroform is a better anæsthetic agent that ether. It may also be regarded as a settled point, that the administration of chloroform is not attended with a sufficient amount of danger to justify, as some think, its rejection altogether. On the other hand, we would not go so far as to say that its use, even with every precaution that science could suggest, is totally devoid of danger. Persons enjoying good health have poisoned themselves with chloroform. Every now and then its administration proves fatal, and no reason can be assigned. But this can be said—for many thousand cases justify the conclusion—that the danger is infinitely small to the advantage gained. The danger, however, exists; hence, he should like to see a third point accepted — viz: that chloroform should not be administered on light occasions, or for trifling ailments, and, above all, that it should not be used except under the superintendence of a competent person. The physiological effects of the agent point out at once the vast range of cases in which it may be employed with benefit. To these he should not allude; but for the present notice a few cases in which, according to his experience, chloroform should not be used. Surgeons are not entirely agreed on these, and it seems more useful to discuss a few doubtful points than to dilate on measures about which no difference of opinion exists. The annihilation of pain is a great victory of chloroform; but, it may be asked, is it always desirable to suspend sensation during surgical operations? He (Mr. Coulson) should say not always. In some rare cases, the feelings of the patient are a useful guide to the surgeon;—hence, in these cases, sensation should not be abolished. This is the principle which should be adopted. As an example of this class of exceptions, lithotrity may be mentioned. It must be confessed that it would be an advantage if chloroform could be employed, as it is a great object to diminish the sensibility of the bladder and the spasm about the neck of that organ which so frequently exist, and not only render the operation in itself difficult, but add to the danger of its results. Yet when the nature of this delicate operation is considered, carried on, as it were, in the dark, and when it is further borne in mind that the operator should be fully aware of every step he is taking, in an organ completely removed from his sight, it cannot be expedient to render the patient insensible, and thus lose the aid which his feelings afford. On many points the patient's sensations are the chief guide which direct the surgeon when he is going wrong, and without them fatal injury might be often inflicted without his being aware of the mischief which he had done. If any operator had arrived at such a degree of dexterity and skill, that it was impossible for him to commit the slightest error, such a man might, perhaps, use chloroform, but until such a man appears, it should not be used. Much mischief might be inflicted unless the feelings of the patient were to control the procedings of the operator. A case in point occurred in a public hospital before the introduction of crushing, at a time when the perforator alone was employed. Notwithstanding the cries of the patient, the surgeon went on using the perforator in a most industrious manner. The bystanders feared something wrong, but he appealed to the sound of a metallic body striking against stone, as a proof that the calculus was actually seized, and undergoing the process of perforation. In a few seconds the cries of the patient became more violent; blood issued abundantly from the urethra. The bystanders now interfered, and pointed out to the surgeon, that the noise which he had heard was produced by the external end of the perforator striking against the seals of his watch-chain. The operation was suspended, and the patient's life saved; but had he been insensible, there is no saying what mischief might not have been inflicted, for the calculus was not between the blades of the instrument. The same principle may, perhaps, be applied to certain obstetric operations, which, like lithotrity, are performed in a deep-seated cavity, and where the feelings of the patient may prevent the infliction of fatal mischief. It often produces vomiting; hence, it should not be used in cases where the effects of vomiting may produce an injurious influence on the health of the patient, or on the results of the operation. As examples, cataract and cleft palate may be mentioned. After division of the cornea for extraction, severe vomiting might cause repulsion of the contents of the eyeball. After sutures of the cleft palate, the severe vomiting may either cause the sutures to give way, or produce such disturbance of the parts as leads to sloughing. Besides this, the active efforts of the patient are required to aid the surgeon in many operations about the fauces; hence another principle of restriction. Indeed, the absence of such active efforts may be a cause of positive danger in certain operations, during which blood may find its way into the air-passages. Some surgeons make light of such accidents, but experience shows that they have often been the cause of much embarrassment, and, at times, of considerable danger. In addition to local circumstances, there may be certain general conditions of the patient which would render the use of chloroform doubtful. Thus it has been a question how far the agent can be safely employed in cases where a severe shock has been already produced by violent and extensive gunshot injuries. Some high authorities condemn the use of chloroform in such cases; while, on the other hand, our younger surgeons in the Crimea apply it without apprehension. It is to be hoped that the opportunities afforded during the present war will enable military surgeons to decide this interesting question. — *Lancet*, October 20, 1855.

